# Chronic Effects of a Training Program Using a Nasal Inspiratory Restriction Device on Elite Cyclists

**DOI:** 10.3390/ijerph18020777

**Published:** 2021-01-18

**Authors:** Jose L. Gonzalez-Montesinos, Jorge R. Fernandez-Santos, Carmen Vaz-Pardal, Ruben Aragon-Martin, Aurelio Arnedillo-Muñoz, Jose Reina-Novo, Eva Orantes-Gonzalez, Jose Heredia-Jimenez, Jesus G. Ponce-Gonzalez

**Affiliations:** 1 Department of Physical Education, Faculty of Education Sciences, University of Cádiz, Puerto Real, 11003 Cádiz, Spain; jgmontesinos@uca.es; 2 Galeno Research Group, Department of Physical Education, Faculty of Education Sciences, University of Cádiz, 11003 Cádiz, Spain; 3 Biomedical Research and Innovation Institute of Cádiz (INiBICA) Research Unit, Puerta del Mar University Hospital University of Cádiz, 11009 Cádiz, Spain; ruben.aragon@gm.uca.es (R.A.-M.); jesusgustavo.ponce@uca.es (J.G.P.-G.); 4 Bahía Sur Andalusian Center for Sports Medicine, 11100 Cádiz, Spain; carmenvaz@hotmail.com; 5 MOVE-IT Research Group, Department of Physical Education, Faculty of Education Sciences, University of Cádiz, 11003 Cádiz, Spain; 6 Allergy and Thoracic Surgery Department, University Hospital Puerta del Mar. Pneumology, 11009 Cádiz, Spain; aurelioarnedillo@neumosur.net; 7 National Cycling Coach, IES, Stadium Path 2504, Hong Kong; jrenovo@hotmail.com; 8 Department of Physical Education & Sports, University of Granada, 18010 Granada, Spain; maevor@ugr.es (E.O.-G.); herediaj@ugr.es (J.H.-J.)

**Keywords:** respiratory muscle training, cyclists, cardiopulmonary exercise testing

## Abstract

This study compared the response of a 9-week cycling training on ventilatory efficiency under two conditions: (i) Combined with respiratory muscle training (RMT) using a new nasal restriction device (FeelBreathe) (FB group) and (ii) without RMT (Control group). Eighteen healthy elite cyclists were randomly separated into the FB group (*n* = 10) or Control group (*n* = 8). Gas exchange was measured breath by breath to measure ventilatory efficiency during an incremental test on a cycloergometer before (Pre) and after (Post) the nine weeks of training. The FB group showed higher peak power (Δ (95%HDI) (0.82 W/kg (0.49, 1.17)), VO_2_max (5.27 mL/kg/min (0.69, 10.83)) and VT_1_ (29.3 W (1.8, 56.7)) compared to Control at Post_FINAL_. The FB group showed lower values from Pre to Post_PRE_ in minute ventilation (VE) (−21.0 L/min (−29.7, −11.5)), Breathing frequency (BF) (−5.1 breaths/min (−9.4, −0.9)), carbon dioxide output (VCO_2_) (−0.5 L/min (−0.7, −0.2)), respiratory equivalents for oxygen (EqO_2_) (−0.8 L/min (−2.4, 0.8)), heart rate (HR) (−5.9 beats/min (−9.2, −2.5)), respiratory exchange ratio (RER) (−0.1 (−0.1, −0.0) and a higher value in inspiratory time (Tin) (0.05 s (0.00, 0.10)), expiratory time (Tex) (0.11 s (0.05, 0.17)) and end-tidal partial pressure of CO_2_ (PETCO_2_) (0.3 mmHg (0.1, 0.6)). In conclusion, RMT using FB seems to be a new and easy alternative ergogenic tool which can be used at the same time as day-to-day training for performance enhancement.

## 1. Introduction

Respiratory muscle training (RMT) has been considered as an effective method to improve the inspiratory muscle strength and performance of athletes of endurance sports [1,2,3,4,5]. Indeed, several studies performed specifically with cyclists have shown that RMT causes physiological adaptations with improvements in the respiratory system, the peak power developed and the time trial performance, both in elite and amateur cyclists [6,7,8,9,10]. However, the RMTs performed in these previous studies have been in static position at rest. Thus, it has not been possible to address the possible additive effect of RMT and exercise at the same time in these previous studies.

Functional RMT while cycling has been investigated previously using 3 different devices: (i) Power Breathe Kinetic KH1 [11]; (ii) Training Mask v2.0 [12]; and (iii) FeelBreathe nasal strips [13]. Using the Power Breathe Kinetic KH1 to RMT while performing stationary cycling increased the electromyography activity in the diaphragm [6]. Despite these results, it should be noted that participants in this study with Power Breathe performed a “static” exercise and, therefore, not a specific cycling training exercise. The use of Training Mask v2.0 while performing 6 weeks of high-intensity cycle ergometer training resulted in improvements in ventilatory threshold, power output at ventilatory threshold, respiratory compensation threshold, and power output at the intensity of respiratory compensation threshold [12]. However, the training mask should be used only part-time during the training season as it could cause inadequate hyperventilation and psychological discomfort [14].

Recently, a new nasal ventilatory flow restriction and filtering device, called FeelBreathe (FB), has been designed, developed and patented to increase nasal airflow resistance [15]. A previous study with elite cyclists has shown that FB used for 10 min on cycle ergometer at 50% of VO_2peak_ causes acute effects in lung ventilation, gas exchange and heart rate during exercise, with improvements on ventilatory efficiency, which could be a target of RMT in sport performance. However, the chronic effect of FB combined with aerobic training in cyclists is unknown [13].

Therefore, the aim of this study was to analyze the additive effect of RMT using FB while performing a specific cycling training plan on different cardiorespiratory variables. We hypothesized, based on the previous results, that the FB group will obtain higher benefits in terms of ventilatory efficiency and peak power developed compared to exercise group without FB without changes on VO_2_ uptake after the period of training.

## 2. Materials and Methods

### 2.1. Subjects

Twenty healthy elite cyclists from two sport clubs in Chiclana de la Frontera (Cádiz, Spain) voluntary participated in this study (mean ± SD, age: 36 ± 10, weight: 71.7 ± 6.7 kg, height: 1.75 ± 0.06 m). All the cyclists had participated in regional and national championships during the last 5 years at least. One of them dropped out the training plan due to illness and another one due to disagreements with the club’s coach, which led to a final sample of eighteen completing the study.

All of the participants were informed of the aims of the study and requirements during the first experimental session. In addition, they signed a written informed consent in accordance with the Declaration of Helsinki. The study protocol and design were approved by the Ethics Committee University Hospital Puerta del Mar (Date: 22 December 2015).

### 2.2. Training Program

The training plan of this study was directed and controlled by a national cycling coach daily. The training intervention lasted 9 weeks, and one group combined the exercise with RMT at the same time as using the FB device (FB group), and another group trained without any airflow restriction (CG group). The training was carried out at the Moreno Periñan velodrome (Chiclana, Cádiz, Spain) and on the road. The distances and slopes of the roads were controlled for the preparation of the training. Physiological evaluations were before (Pre) and after (Post) the training program to evaluate breathing efficiency through gas exchange.

**Figure 1:**
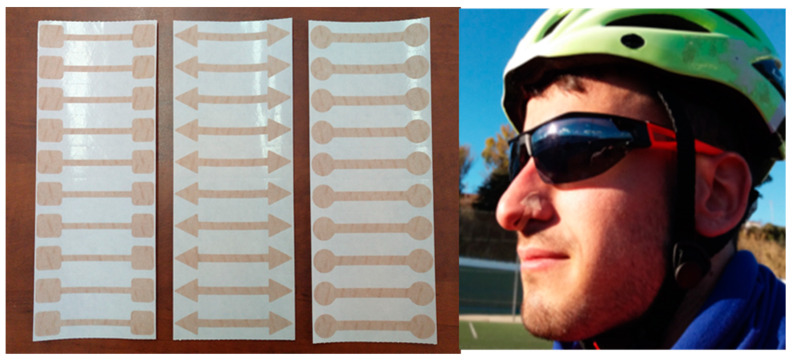
FeelBreathe (FB) devices of 4, 5 and 6 mm arranged in sheets of 10 units. FB placement mode under the nostrils.

FB was manufactured for the present study in three models, 4, 5 and 6 mm of ventilatory flow restriction, which produce different levels of air restriction and inspiratory effort. FB has been authorized by the Spanish Agency for Medicines and Health Products (AEMPS No. Exp: 521/15/EC, Spain) (Figure 1). The use of this device during exercise has been used in previous investigations, both in athletes [13] and in patients with COPD [16,17].

Participants were randomly assigned to either the cycling training combined with FB (FB, *n* = 10) or control group without FB (Control, *n* = 8). Both groups were matched by age and VO_2_max. All participants were instructed to avoid changes in their diet or physical activity while they were following the training plan. The training intensity was set based on the heart rate corresponding to the lactate threshold (HRlactate): (i) Regenerative (<75% HRlactate); A0 (75–90% HRlactate); A1 (90–95% HRlactate); A2 (95–100% HRlactate); A3 (100–105% HRlactate); A4 (105% HRlactate—HRpeak). Both groups completed a volume of 144 h of training during the 9 weeks of intervention, with similar intensity and duration adapted to each athlete (Regenerative: ~42/43% of total training time, A0: ~36/38%, A1: ~8/9%, A2: ~8%, A3: ~3%, A4: ~1%).

The participants of the FB group were instructed on how to place the FB device correctly. During the first 2 weeks, all the participants used the 4 mm FB device model, increasing the width of the device to the 5 mm FB model for the next 4 weeks and to 6 mm FB during the last 3 weeks of training.

### 2.3. Measurements

At the Andalusian Center for Sports Medicine (Bahía Sur, San Fernando, Spain), pre and post training tests were performed on all participants. During the testing day, resting tests were done with measurement of weight and height, blood pressure, cardiopulmonary auscultation, baseline spirometry (Cardinal Health Spirometer, D-97204 Hoechberg, Germany) and a twelve-lead resting electrocardiogram (Mortara R-SCRIBETM 5, Milwaukee, USA). Moreover, maximal inspiratory pressure (MIP) (Micro RPM of Micro Medical, Chatham, Kent, UK) was measured according to American Thoracic Society (ATS) protocol, choosing the highest value of the 3 efforts with a lower than 5% difference [18].

After that, all participants completed an incremental test on a cycle ergometer (Cardgirus Bikemarc SL, Barcelona, Spain). This test consisted of a stepwise protocol of 30 watt increments every minute with constant revolutions per minute of 80–85 rpm, as used previously [19,20]. The incremental test continued until exhaustion or until the cadence was not maintained. Gas exchange and ventilatory variables were recorded during the incremental test using a gas analyzer (Jaeger-CareFusion modelo MasterScreen CPX). The oxygen consumption (VO_2_), carbon dioxide output (VCO_2_), Breathing frequency (BF), tidal volume (Vt), inspiratory tidal volume (VTin), expiratory tidal volume (VTex), inspiratory time (Tin), expiratory time (Tex), duty cycle (TiTot), minute ventilation (VE), respiratory equivalents for oxygen (EqO_2_) and carbon dioxide (EqCO_2_), end-tidal partial pressure of O_2_ (PETO_2_) and CO_2_ (PETCO_2_), and respiratory exchange ratio (RER) were registered in a breath-by-breath manner and averaged by 10 s. Moreover, two dependent evaluators assessed VT_1_ from visual inspection of (1) the first disproportionate increase in VE; (2) an increase in VE/VO_2_ with no increase in VE/VCO_2_ (i.e., the ventilatory equivalents); (3) an increase in PETO_2_ with no consequent fall in PETCO_2_, and (4) V-slope plot. VT_2_ was determined from visual inspection of (1) the second disproportionate increase in VE, (2) the first systematic decrease in PETCO_2_, and (3) the first systematic increase in VE/VCO_2_.

Rating of Perceived Exertion (RPE) was detected using the Borg Scale CR 0–10 [21]. One minute after the end of exercise, MIP were repeated post-exercise. After the 9-week training program, all post-tests were performed in the same order and conditions of the pre-test. The evaluators who performed the tests were blind to which individual they were evaluating since they did not participate in the training sessions.

## 3. Statistical Analysis

**Figure 2:**
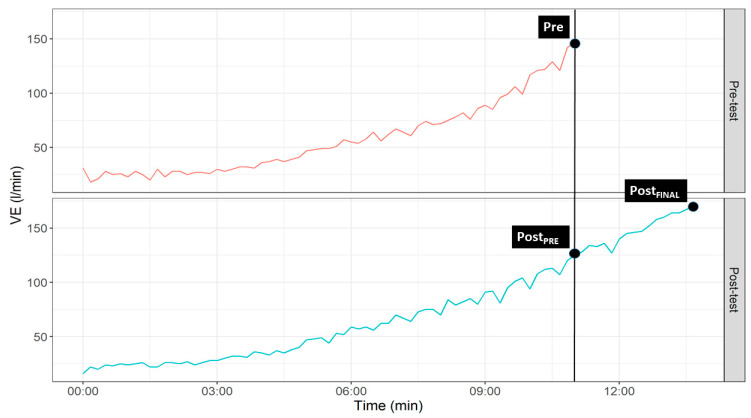
Graphical representation of temporal points selected for statistical analyses.

Values are reported as mean ± standard deviation. For cardiorespiratory variables analyses, three values were obtained corresponding to three different temporal points in the incremental test: The maximum value recorded at the pre-training test (Pre), the value obtained at the post-training test at the same time as the maximum value at the pre-training test (Post_PRE_) was obtained, and the maximum value recorded at the post-training test (Post_FINAL_) (Figure 2).

Within- and between-group differences were assessed using a Bayesian hierarchical regression model. All hyperparameters in the model followed a weakly informative prior distribution (i.e., a prior distribution that encoded enough information to restrict the plausible range of values of the parameter space but still left a wide range of values to be covered) [22]. Inference was performed based on the 95% highest density interval (95%HDI), which contains a range of values where we can be 95% certain that the true value lies given the data at hand and the model fitted. The null value in our analysis is 0, so if this number is not inside the 95%HDI then we can reject that value for practical purposes [23]. Bayesian estimation of the parameters was obtained by using the package *brms* for the R programming language [24]. All parameters estimated showed a good convergence with values of $\hat{R}$ = 1 and number of effective sample size > 1000. Further details about the analysis can be found in the supplemental file S1, while the code and the dataset to replicate it are stored in https://github.com/JorgeDelro/cyclists_PE.

## 4. Results

No significant differences were found between FB and CG groups at the baseline (Table 1).

Regarding within-group differences, the FB group (Table 2) obtained a lower value from Pre to Post_PRE_ in VE (Δ (95%HDI) = −21.0 L/min (−29.7, −11.5)), BF (−5.1 breaths/min (−9.4, −0.9)), VCO_2_ (−0.5 L/min (−0.7, −0.2)), EqO_2_ (−0.8 L/min (−2.4, 0.8)), HR (−5.9 beats/min (−9.2, −2.5)) and RER (−0.1 (−0.1, −0.0) and a higher value in Tin (0.05 s (0.00, 0.10)), Tex (0.11 s (0.05, 0.17)) and PETCO_2_ (0.3 mmHg (0.1, 0.6)). Additionally, the FB group (Table 2) increased the peak power from Pre to Post_FINAL_ (0.7 W/kg (0.5, 0.9)), VT_1_ (21.0 W (9.6, 32.4)) and VT_2_ (17.7 W (0.7, 36.2)). The Control group (Table 3) reached a lower value from Pre to Post_PRE_ in VCO_2_ (−0.2 L/min (−0.5, −0.0)) and HR (−4.6 beats/min (−7.7, −1.1)) and a higher value in VT_2_ (21.0 W (9.6, 32.4)).

Between-group differences for breathing conditions (Table 4) showed a difference in VT_2_ (7.69 mL/kg/min (1.86, 13.27)) at Pre; in BF (−10.73 breath/min (−19.7, −2.13)), tin (0.10 s (−0.00, −0.20)), tex (0.19 s (0.09, 0.30)) and PETCO_2_ (0.56 mmHg (0.15, 0.97)) at Post_PRE_; in Peak power (0.82 W/kg (0.49, 1.17)), VO_2_max (5.27 mL(kg/min (0.69, 10.83)) and VT_1_ (29.3 W (1.8, 56.7)) at Post_FINAL_; in VCO_2_ (−371.7 L/min (−732.9, −10.2)), tex (0.14 s (0.01, 0.27)), titot (−2.49% (−4.10, −0.85)) and PETO_2_ (−0.51 mmHg (−0.99, −0.03)) of Δ at Post_PRE_; in Peak power (0.58 W/kg (0.23, 0.92)) and HR (5.0 beats/min (3.5, 9.6)) of Δ at Post_FINAL_.

Within-group differences were found pre-test and pre-training vs. pre-test and post-training in MIP for FB (30.5 cmH_2_O (18.1, 43.0)) and CG (15.4 cmH_2_O (2.6, 27.7) and in RPE post-training vs. pre-training for the control group (0.7 (0.1–1.4)) (Table 5).

Finally, no significant between-group differences were found in the increments in MIP or RPE values (Table 6).

## 5. Discussion

The main finding of the present study was that the ventilatory efficiency and the breathing pattern were improved after the exercise training program in the FB group at the maximum intensity reached in the Pre-test (Post_PRE_) but not in the control group. FB showed improvements in the time trial and hence in the maximum peak of power developed in the maximum test but without changes in the VO_2_max compared to pre-training values, while the Control group remained unchanged. This could be explained in part by the higher VO_2_max and VT_1_ in the FB compared to the Control group after the training program at maximum values (Post_FINAL_). Moreover, VT_2_ showed similar values between conditions after intervention despite the Control group beginning with higher values at Pre. Therefore, only the combination of cyclist training with RMT and FB optimized the breathing pattern such that it could improve performance. To our knowledge, this is the first study which has analyzed the benefits on ventilatory efficiency and breathing pattern in elite cyclists after 9 weeks of traditional endurance training combined with respiratory muscle training at the same time as FB is used.

In agreement with our results, RMT has been documented to improve performance in a wide range of exercise modalities including running, cycling, swimming and rowing [3]. In a study by Holm et al. [7], where 20 trained cyclists and triathletes underwent aerobic training of the respiratory muscles, a significant improvement (of 4.75%) was observed in the time trial as well as in the endurance of the respiratory muscles compared to the control group, but without changes in VO_2_max. In agreement, McEntire et al. [25], showed that specific training of respiratory muscles through the use of Power Breathe at 15% of MIP in cyclists improved physical performance (18% Exercise Group vs. 10% control group), also without changes in VO_2_max, RPE or dyspnea. In that sense, our study found that the FB group improved 14.3% in the time trial after intervention, while the Control group remained unchanged (~3%) and without any effect on VO_2_max in both groups.

The observed gain in the time trial performance in these studies, without an increase in VO_2_max, may be due to an improvement on ventilatory efficiency. In concordance, our results showed improvements only in FB group at Post_PRE_ moment with significant reductions in VE, BF, VCO_2_, EqO_2_, Vt/Ti and RER values and with significant increments in Tin, Tex and PETCO_2_ values at the same maximum intensity of Pre-moment.

Minute VE was reduced by 13.8% in a moderate intensity exercise in hypoxic condition after only four weeks of inspiratory muscle training (IMT), which means a reduction of the physiological demand of exercise [26]. Similar reduction in VE (15%) was observed only in the FB group in our study at the Post_PRE_ moment. Moreover, it was only in the FB group that the BF dropped ~12%, which can indicate, together with a longer inspiratory time (~9.8%), that there has been a correct training of the respiratory muscles, which are then able to obtain slower and deeper breaths. This, together with a reduction in EQO_2_ of −8.6% without changes in EQCO_2_, leads us to a lower dynamic hyperinflation and greater ventilatory efficiency with a similar oxygen uptake with lower ventilation. This physiologic phenomenon triggered a lower heart frequency (~3.5%) and RER (~6.6%) at the same intensity moment, therefore showing an improvement in cardiovascular performance, lower cardiac output and higher energy efficiency. Moreover, FB causes nasal inspirations and mouth exhalations during training sessions, improving the humidification, heating and filtering of the air as it represents a normal mechanism of heat and moisture exchange in the respiratory tract [27].

All changes on ventilatory parameters in FB group after the intervention lead to the improvement of both VT_1_ and VT_2_ thresholds (~13.2 and 6.3%; respectively), which are relevant factors for performance and metabolic flexibility during exercise. In fact, a shift to the right of ventilatory thresholds means a better use of fat as an energy source, saving energy from muscle glycogen, preventing fatigue and improving the trial time and the peak of power developed. Despite the fact that the FB group improved the peak of maximum power after training, the subjective perception of effort remained unchanged. Hence, RMT with FB combined with aerobic training may improve the efficiency of oxygen delivery, transport and utilization for fat oxidation during exercise. The group that trained without FB only improved the VT_2_, but this could be due in part to the fact that this group begun the intervention with lower values in Pre-moment compared to the FB group. However, the magnitude of the Pre-Post change was similar for VT_2_ between groups, but not for VT_1_, where the FB group obtained greater improvements.

Furthermore, one of the most important variables to quantify respiratory performance is the MIP. Enright el al. [28] showed that, after 8 weeks of IMT, there was a significant increase in MIP from 142 to 193 cmH_2_O. In agreement, another study conducted with cyclists demonstrated that IMT for 10 weeks improved MIP by 34% and test time to exhaustion [29]. Moreover, Archiza et al. [30] found that 6 weeks of pressure-threshold IMT improved running time to exhaustion and repeated sprint ability in soccer players. In our study, the FB group showed a significant increase in MIP of 30.5 cmH_2_O, while that of the CG group was only 15.4 cmH_2_O. However, no significant between-groups differences were found in the increments of MIP. Moreover, the FB group started from a lower average baseline, which may be responsible for the steeper increase. Nonetheless, the higher effect found in MIP at Pre vs. Pre and the positive change showed from pre-test to post-test either pre- and post-training (negative for CG group) make us claim that further studies are necessary to confirm the ergogenic effect of FB on MIP.

Thus, RMT has been proposed as an ergogenic aid for performance enhancement in training protocols [3]. In this sense, recent studies have been shown the positive effect of training the respiratory muscles at the same time as exercise [12] and not doing so in static situations as is usually done [1,31]. This is, without doubt, one of the biggest advantages of FB.

## 6. Conclusions

FB is a new and easy device for respiratory muscle training that can be used during the practice of physical exercise. It also could be used while performing most daily tasks unlike other devices for IMT, which have to be used in static position. Moreover, FB is a valid and useful alternative to the training mask since it could be easier and, especially, more comfortable. Hence, FB could be incorporated into the training of this type of athlete as a further stimulus to training with the goal of improving both the specific and respiratory muscles.

## Figures and Tables

**Table 1:** Participant’s baseline characteristics by group.

Variable	FB (*n* = 10)	CG (*n* = 8)	FB vs. CG
Age (years)	35.3 ± 9.4	38.1 ± 11.7	−1.4 (−6.2, 3.9)
Weight (kg)	71.2 ± 4.0	72.2 ± 9.4	−0.5 (−3.7, 2.9)
Height (cm)	176.8 ± 7.1	172.5 ± 4.9	2.0 (−1.1, 5.0)
BMI (kg/m^2^)	22.7 ± 2.0	24.3 ± 2.9	−0.7 (−1.8, 0.6)
VO_2max_ (mL/kg/min)	55.8 ± 4.7	51.1 ± 5.4	2.3 (−0.2, 4.6)
MIP (cmH_2_O)	155.5 ± 36.2	167.6 ± 51.7	−5.4 (−27.7, 14.8)

FB, Feelbreathe group; CG, control group. Values are expressed as mean ± standard deviation. Differences are expressed as mean (95% HDI).

**Table 2:** Average of the maximum value obtained by the Feelbreathe group (FB) in each variable at the pre-training test (Pre-Value), value obtained in the post-training test at the same moment that the maximum in the pre-training test was obtained (Post_PRE_-Value), and maximum obtained at the post-training test (Post_FINAL_-Value). Percentage of change (%_Change_), increment (Δ), and 95% HDI are reported from Pre to Post_PRE_ and from Pre to Post_FINAL_.

FB (*n* = 10)							
	Pre	Post_PRE_			Post_FINAL_		
Variable	Value	Value	%_Change_	Δ (95%CrI)	Value	%_Change_	Δ (95%CrI)
Power (W/kg)	4.9 ± 0.3	5.0 ± 0.4	3.3	0.2 (−0.1, 0.4)	**5.6 ± 0.4**	**14.3**	**0.7 (0.5, 0.9)**
VO_2_max (mL/min)	3981.4 ± 475.8	3714.2 ± 425.2	−6.7	−205.0(−416.0, 16.7)	3988.3 ± 479.6	0.2	11.1 (−217.0, 225.0)
VO_2_max (mL/kg/min)	55.8 ± 4.7	53.2 ± 5.8	−4.7	−2.5 (−6.1, 1.6)	56.8 ± 6.6	1.8	0.8 (−3.3, 4.7)
VT_1_ (W)	159.0 ± 28.5				**180.0 ± 33.9**	**13.2**	**21.0 (9.6, 32.4)**
VT_1_ (mL/kg/min)	26.7 ± 3.9				**29.6 ± 5.7**	**10.8**	**2.9 (0.5, 5.4)**
VT_1_ (L/min)	1.9 ± 0.4				**2.1 ± 0.5**	**10.5**	**0.2 (0.0, 0.4)**
VT_2_ (W)	288.0 ± 28.1				**306.0 ± 30.2**	**6.3**	**17.7 (0.6, 36.2)**
VT_2_ (mL/kg/min)	47.1 ± 8.7				46.7 ± 8.0	−0.8	−0.4 (−0.4, 3.8)
VT_2_ (L/min)	3.4 ± 0.4				3.3 ± 0.6	−0.9	−0.0 (−0.3, 0.3)
VE (L/min)	149.1 ± 24.0	**126.6 ± 21.4**	**−15.1**	**−21.0 (−29.7, −11.5)**	152.0 ± 15.7	1.9	3.6 (−12.5, 18.8)
BF (breaths/min)	49.4 ± 10.5	**43.4 ± 10.0**	**−12.1**	**−5.1 (−9.4, −0.9)**	51.5 ± 7.2	4.3	2.9 (−3.6, 9.7)
VCO_2_ (L/min)	5.2 ± 0.5	**4.5 ± 0.3**	**−13.5**	**−0.5 (−0.7, −0.2)**	4.9 ± 0.3	−5.8	−0.2 (−0.4, 0.1)
EqO_2_ (L/min)	36.2 ± 4.4	**33.1 ± 6.3**	**−8.6**	**−3.4 (−5.8, −0.7)**	37.9 ± 4.9	4.7	0.8 (−2.3, 3.7)
EqCO_2_ (L/min)	27.7 ± 2.4	26.9 ± 3.2	−2.9	−0.8 (−2.4, 0.8)	29.7 ± 3.2	7.2	2.1 (−0.3, 4.4)
HR (beats/min)	181.2 ± 8.9	**174.9 ± 9.8**	**−3.5**	**−5.9 (−9.2, −2.5)**	183.3 ± 7.1	1.2	2.0 (−1.0, 5.2)
VTin (L)	3.1 ± 0.3	2.7 ± 1.0	−12.9	−1.2 (−0.3, 0.1)	3.04 ± 0.25	−1.3	−0.04 (−0.22, 0.16)
VTex (L)	3.1 ± 0.3	2.6 ± 1.0	−16.1	−0.4 (−0.9, 0.1)	2.97 ± 0.20	−2.6	−0.10 (−0.32, 0.14)
Tin (s)	0.61 ± 0.11	**0.67 ± 0.14**	**9.8**	**0.05 (0.00, 0.10)**	0.56 ± 0.08	−8.2	−0.05 (−0.18, 0.03)
Tex (s)	0.64 ± 0.11	**0.77 ± 0.15**	**20.3**	**0.11 (0.05, 0.17)**	0.62 ± 0.11	−3.1	−0.03 (−0.12, 0.05)
TiTot (%)	48.5 ± 1.9	46.4 ± 2.4	−4.3	−2.1 (−3.2, 0.9)	47.7 ± 2.2	−1.6	−0.1 (−0.6, 0.4)
PETO_2_ (mmHg)	16.0 ± 0.4	15.4 ± 0.7	−3.8	−0.4 (−0.63, −0.15)	15.9 ± 0.2	−0.6	−0.0 (−0.3, 0.2)
PETCO_2_ (mmHg)	4.8 ± 0.4	**5.2 ± 0.5**	**8.3**	**0.3 (0.1, 0.6)**	4.8 ± 0.5	0.6	0.02 (−0.3, 0.3)
RER	1.31 ± 0.09	**1.22 ± 0.13**	**−6.9**	**−0.1 (−0.1, −0.0)**	1.25 ± 0.10	−4.6	−0.06 (−0.11, −0.01)

95% HDIs that 0 is not inside are expressed in bold.

**Table 3:** Average of the maximum value obtained by the control group (CG) in each variable at the pre-training test (Pre-Value), value obtained in the post-training test at the same moment that the maximum in the pre-training test was obtained (Post_PRE_-Value), and maximum obtained at the post-training test (Post_FINAL_-Value). Percentage of change (%_Change_), increment (Δ), and 95% HDI are reported from Pre to Post_PRE_ and from Pre to Post_FINAL_.

CG (*n* = 8)							
	Pre	Post_PRE_			Post_FINAL_		
Variable	Value	Value	%_Change_	Δ (95%CrI)	Value	%_Change_	Δ (95%CrI)
Power (W/kg)	4.6 ± 0.3	4.7 ± 0.3	0.9	0.1 (−0.2, 0.3)	4.8 ± 0.3	3.0	0.1 (−0.1, 0.4)
VO_2_max (mL/min)	3673.0 ± 451.8	3581.6 ± 508.5	−2.5	−94.5(−326.0, 125.0)	3689.3 ± 491.3	0.4	8.1 (−218.0, 239.0)
VO_2_max (mL/kg/min)	51.1 ± 5.4	49.4 ± 3.9	−3.3	−1.6 (−5.6, 2.7)	50.9 ± 4.0	−0.4	−0.1 (−4.6, 4.2)
VT_1_ (W)	144.4 ± 28.8				150.6 ± 27.8	4.3	6.2 (−7.1, 20.3)
VT_1_ (mL/kg/min)	23.6 ± 4.8				25.9 ± 3.6	9.8	2.3 (−0.5, 5.2)
VT_1_ (L/min)	1.7 ± 0.4				1.9 ± 0.4	9.9	0.2 (−0.0, 0.4)
VT_2_ (W)	253.8 ± 52.7				**286.3 ± 38.1**	**12.8**	**32.5 (11.4, 53.7)**
VT_2_ (mL/kg/min)	39.1 ± 6.5				44.1 ± 5.4	12.8	5.0 (0.6, 9.4)
VT_2_ (L/min)	2.8 ± 0.6				**3.2 ± 0.6**	**13.1**	**0.3 (0.1, 0.7)**
VE (L/min)	141.9 ± 17.4	139.6 ± 17.9	−1.6	−1.8 (−11.9, 7.70)	140.5 ± 22.3	−1.0	−1.9 (−19.0, 15.7)
BF (breaths/min)	53.3 ± 8.7	54.8 ± 9.3	2.8	1.6 (−3.3, 6.2)	53.0 ± 7.5	−0.6	0.1 (−7.0, 6.4)
VCO_2_ (L/min)	4.8 ± 0.7	**4.5 ± 0.6**	**−6.3**	**−0.2 (−0.5, −0.0)**	4.9 ± 0.3	2.1	−0.1 (−0.4, 0.1)
EqO_2_ (L/min)	37.2 ± 3.9	37.3 ± 2.3	0.2	0.4 (−2.3, 3.2)	36.4 ± 1.9	−2.2	−0.5 (−2.3, 3.7)
EqCO_2_ (L/min)	28.7 ± 2.8	29.4 ± 1.3	2.4	1.2 (−0.7, 2.8)	29.0 ± 2.8	1.0	0.5 (−2.3, 3.0)
HR (beats/min)	178.4 ± 12.6	**173.8 ± 13.5**	**−2.6**	**−4.6 (−7.7, −1.1)**	175.4 ± 14.8	−1.7	−3.0 (−6.4, 0.4)
VTin (L)	2.74 ± 0.57	2.69 ± 0.6	−1.8	−0.03(−0.29, 0.24)	2.75 ± 0.56	0.4	0.01 (−0.20, 0.24)
VTex (L)	2.73 ± 0.60	2.62 ± 0.59	−4.0	−0.1 (−0.7, 0.5)	2.70 ± 0.54	−1.1	−0.02 (−0.27, 0.22)
Tin (s)	0.57 ± 0.11	0.56 ± 0.10	−1.8	−0.01 (−0.06, 0.04)	0.57 ± 0.09	0.5	0.01(−0.07, 0.09)
Tex (s)	0.58 ± 0.10	0.57 ± 0.09	−1.7	−0.01 (−0.08, 0.05)	0.58 ± 0.06	0.5	−0.01 (−0.09, 0.08)
TiTot (%)	49.3 ± 2.0	49.6 ± 1.6	0.6	0.4 (−0.8, 1.5)	49.9 ± 2.2	1.2	−0.0 (−0.3, 0.3)
Vt/Ti (L/s)	4.81 ± 0.56	4.70 ± 0.64	−2.3	−0.1 (−0.3, 0.2)	4.72 ± 0.82	−1.9	−0.1 (−0.6, 0.4)
Vt (L)	2.74 ± 0.61	2.62 ± 0.60	−4.4	−0.1 (−0.3, 0.1)	2.69 ± 0.54	−1.8	−0.0 (−0.3, 0.2)
PETO_2_ (mmHg)	16.1 ± 0.3	16.1 ± 0.3	−0.1	−0.1 (−0.31, 0.1)	16.0 ± 0.2	−0.6	−0.1 (−0.3, 0.1)
PETCO_2_ (mmHg)	4.7 ± 0.4	4.6 ± 0.1	−2.1	0.1 (−0.2, 0.3)	4.8 ± 0.5	2.2	0.02 (−0.21, 0.26)
RER	1.30 ± 0.05	1.27 ± 0.10	−2.3	−0.0 (−0.1, 0.0)	1.26 ± 0.09	−3.1	−0.04 (−0.09, 0.02)

95% HDIs that 0 is not inside are expressed in bold.

**Table 4:** Between-group differences for breathing conditions at the pre-training test (Pre), at the value obtained in the post-training test at the same moment that the maximum in the pre-training test was obtained (Post_PRE_), at the maximum value obtained at the post-training test (Post_FINAL_), and between increments at Post_PRE_ (Δ Post_PRE_) and Post_FINAL_ (Δ Post_FINAL_).

Variable	FB vs. Control				
	Pre	Post_PRE_	Post_FINAL_	Δ Post_PRE_	Δ Post_FINAL_
Power (W/kg)	0.26 (−0.09, 0.59)	0.39 (−0.04, 0.76)	**0.82 (0.49, 1.17)**	0.12 (−0.18, 0.44)	**0.58 (0.23, 0.92)**
VO_2_max (mL/min)	285.0 (−131.0, 750.0)	172 (−277.0, 619.0)	280.0 (−157.0, 740.0)	−110.6 (−366.0, 152.0)	−5.1 (−362.9, 346.8)
VO_2_max (mL/kg/min)	4.53 (−0.19, 9.45)	4.27 (−1.0, 9.0)	**5.27 (0.69, 10.83)**	−0.39 (−5.12, 4.35)	1.2 (−4.5, 6.9)
VT_1_ (W)	14.5 (−12.4, 43.0)		**29.3 (1.8, 56.7)**		14.8 (−3.5, 32.9)
VT_1_ (mL/kg/min)	2.94 (−1.26, 7.31)		3.58 (−0.68, 7.93)		0.5 (−3.2, 4.3)
VT_1_ (L/min)	0.20 (−0.18, 0.60)		0.23 (−0.13, 0.63)		0.03 (−0.22, 0.29)
VT_2_ (W)	32.5 (−5.17, 67.5)		19.4 (−17.5, 54.7)		−14.8 (−43.6, 13.9)
VT_2_ (mL/kg/min)	**7.69 (1.86, 13.27)**		2.61 (−2.77, 8.43)		−5.4 (−11.3, 0.6)
VT_2_ (L/min)	0.51 (−0.00, 0.98)		0.13 (−0.36, 0.61)		0.2 (−0.8, 0.0)
VE (L/min)	6.68 (−12.25, 26.0)	−12.01 (−31.76, 6.1)	11.48 (−7.25, 30.8)	−19.77 (−39.95, 0.10)	5.5 (−18.9, 30.2)
BF (breaths/min)	−3.90 (−11.9, 4.88)	**−10.73 (−19.7, −2.13)**	−1.51 (−10.3, 7.21)	−7.20 (−17–41, 3.19)	2.8 (−6.5, 11.3)
VCO_2_ (L/min)	−382.6 (−889, 82.4)	−22.1 (−542, 436.0)	−287.7 (−772, 223.8)	**−371.7 (−732.9, −10.2)**	−0.03 (−0.30, 0.24)
EqO_2_ (L/min)	−1.03 (−4.97, 3.15)	−4.36 (−8.41, 0.03)	0.51 (−3.84, 4.55)	−3.51 (−8.07, 1.21)	1.3 (−3.2, 5.8)
EqCO_2_ (L/min)	−0.96 (−3.57, 1.44)	−2.50 (−4.97, 0.38)	0.65 (−1.87, 3.09)	−1.61 (−4.87, 1.81)	1.6 (−1.8, 5.2)
HR (beats/min)	2.80 (−6.91, 13.1)	1.46 (−8.96, 11.3)	7.78 (−2.58, 17.5)	−1.08 (−5.47, 3.27)	**5.0 (3.5, 9.6)**
VTin (mL)	320.7 (−177.0, 858.0)	−21.3 (−530.0, 564.0)	4.1 (−536.0, 522.0)	−371.0 (−1075.3, 335.0)	−0.05 (−0.34, 0.24)
Vtex (mL)	340.3 (−174.0, 880.0)	−28.6 (−586.0, 509.0)	−9.8 (−543.0, 518.0)	−324.0 (−1002.6, 374.1)	−0.07 (−0.41, 0.26)
Tin (s)	0.03 (−0.06, 0.13)	**0.10 (−0.00, −0.20)**	−0.01 (−0.11, 0.08)	0.06 (−0.04, 0.17)	−0.05 (−0.15, 0.06)
Tex (s)	0.01 (−0.04, 0.16)	**0.19 (0.09, 0.30)**	0.04 (−0.06, 0.14)	**0.14 (0.01, 0.27)**	−0.02 (−0.14, 0.09)
Titot (%)	−0.79 (−2.85, 1.07)	−3.19 (−5.25, 1.10)	−2.12 (−4.07, 0.02)	**−2.49 (−4.10, −0.85)**	−0.1 (−0.5, 0.3)
PETO_2_ (mmHg)	−0.17 (−0.58, 0.27)	−0.65 (−1.08, 0.21)	−0.06 (−0.46, 0.37)	**−0.51 (−0.99, −0.03)**	0.0 (−0.2, 0.3)
PETCO_2_ (mmHg)	0.11 (−0.28, 0.50)	**0.56 (0.15, 0.97)**	0.06 (−0.36, 0.46)	0.48 (−0.02, 0.98)	0.00 (−0.28, 0.30)
RER	0.01 (−0.07, 0.09)	−0.05 (−0.14, 0.04)	−0.00 (−0.09, 0.09)	−0.06 (−0.12, 0.01)	−0.02 (−0.09, 0.05)

95% HDIs that 0 is not inside are expressed in bold.

**Table 5:** Within-group pre- and post-test differences in MIP and RPE values.

**FB (*n* = 10)**					
Variable	Pre-Incremental Test	Post-Incremental Test	%_Change_	Δ (95% HDI)	
				Pre vs. Pre	Δ_POST_ vs. Δ_PRE_
MIP_PRE_ (cmH_2_O)	165.3 ± 35.5	172.4 ± 34.4	4.3	**30.5** **(18.1, 43.0)**	−7.5 (−24.7, 10.2)
MIP_POST_ (cmH_2_O)	198.2 ± 35.2	200.9 ± 36.7	1.3		
	Value		%_Change_	Post vs. Pre	
RPE_PRE_	8.9 ± 1.0		6.7	0.6 (−0.0 to 1.2)	
RPE_POST_	9.5 ± 0.5				
**CG (*n* = 8)**					
Variable	Pre-incremental test	Post- incremental test	%_Change_	Δ (95% HDI)	
				Pre vs. Pre	Δ_POST_ vs. Δ_PRE_
MIP_PRE_ (cmH_2_O)	180.8 ± 44.0	178.5 ± 54.7	−1.3	**15.4** **(2.6, 27.7)**	3.7 (−14.7, 21.9)
MIP_POST_ (cmH_2_O)	195.6 ± 37.1	191.6 ± 33.0	−2.1		
	Value		%_Change_	Post vs. Pre	
RPE_PRE_	8.4 ± 0.9		9.5	**0.7 (0.1, 1.4)**	
RPE_POST_	9.2 ± 0.7				

95% HDIs that 0 is not inside are expressed in bold. MIP: maximal inspiratory pressure; RPE: rating of perceived exertion; _PRE_ and _POST_ subindex indicates pre- and post-training respectively; %_Change_, percentage of change; Pre vs. Pre, pre-test and pre-training MIP value vs. pre-test and post-training MIP value; Δ_POST_ vs. Δ_PRE_, increase in MIP value during incremental test post-training vs. increase in MIP value during PE test pre-training; Post vs. Pre, post-training RPE value vs. pre-training RPE value.

**Table 6:** Between-group differences in the increments in MIP and RPE values.

Variable		Δ (95% HDI)	
	Pre vs. Pre	Δ_POST_ vs. Δ_PRE_	Post vs. Pre
MIP (cmH_2_O)	17.0 (−1.6, 35.3)	−4.5 (−22.3, 10.7)	
RPE			−1.1 (−2.4, 0.2)

Differences are expressed as FB vs. CG. MIP: maximal inspiratory pressure; RPE: rating of perceived exertion. Pre vs. Pre, pre-test and pre-training MIP value vs. pre-test and post-training MIP value; Δ_POST_ vs. Δ_PRE_, increase in MIP value during incremental test post-training vs. increase in MIP value during PE test pre-training; Post vs. Pre, post-training RPE value vs. pre-training RPE value.

## Data Availability

The data is stored in https://github.com/JorgeDelro/cyclists_PE.

## Supplementary Materials

The following are available online at https://www.mdpi.com/1660-4601/18/2/777/s1. File S1: Effect of a training programme using a nasal inspiratory restriction device in elite cyclists.
