# Supplementary material for: Chronic Effects of a Training Program Using a Nasal Inspiratory Restriction Device on Elite Cyclists
Source: Int J Environ Res Public Health. 2021 Jan 18;18(2):777. doi: 10.3390/ijerph18020777 (PMC7831303; doi:10.3390/ijerph18020777)
Supplement: Supplementary file 1 [file ijerph-18-00777-s001.pdf]

SUPPLEMENTAL FILE

EFFECT OF A TRAINING PROGRAMME USING A NASAL INSPIRATORY RESTRICTION DEVICE IN ELITE CYCLISTS

1. Bayesian data analysis

All the variables were analyzed following the same workflow: i) graphical data exploration, ii) model definition; iii) prior predictive checking; iv) parameter estimation and graphical diagnostics and v) posterior predictive checking. Here we show the analysis of VE as example.

1.1. Graphical data exploration

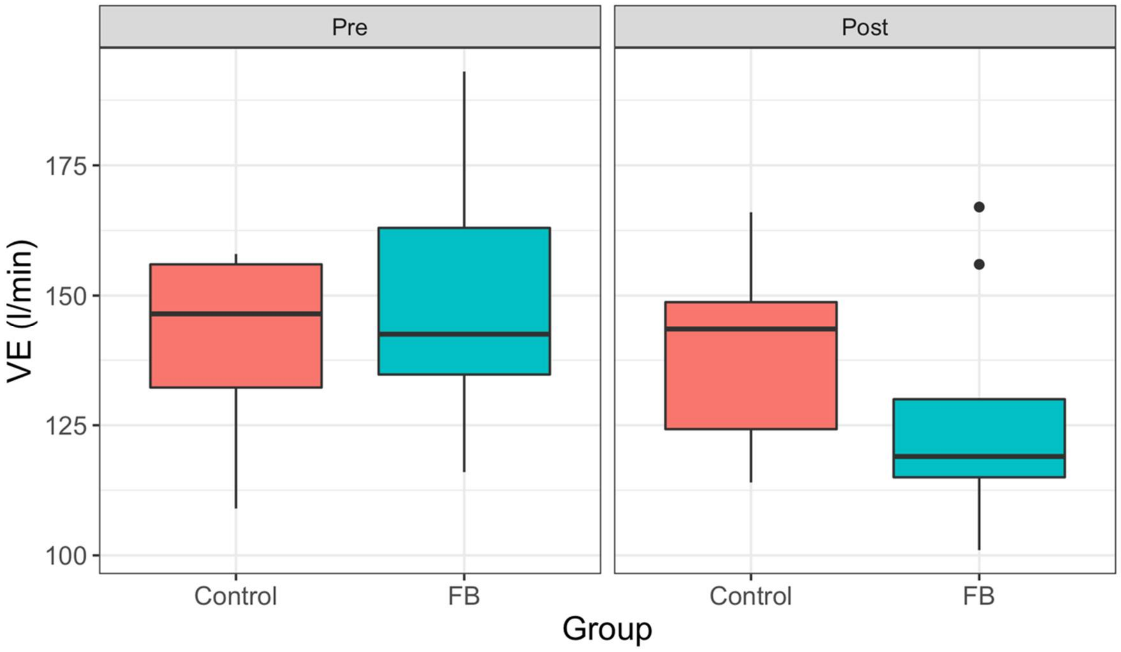

Figure S1. Boxplot of VE for Feelbreathe(FB) and Control group at pre-training (Pre) and post-training (Post).

1.2. Model definition

$VE_i \sim$ StudentT(u_i, σ, υ) [likelihood]

$u_i = \alpha_{[ID]} + \beta_1$ Group$_j$ + β_2 Time$_t$ + β_3 Group$_j$ Time$_t$ [linear model]

$\sigma \sim$ half-StudentT(0, 26.7, 3) [residual standard deviation]

$\upsilon \sim$ Gamma(2, 0.1) [degrees of freedom]

$\beta_1 \sim$ Normal(0, 54) [weakly informative prior]

$\beta_2 \sim$ Normal(0, 54) [weakly informative prior]

$\beta_3 \sim$ Normal(0, 54) [weakly informative prior]

$\alpha_{[ID]} \sim$ Normal (α, σ_α) [adaptive prior]

$\alpha \sim$ StudentT(138, 26.7, 3) [prior for average ID]

$\sigma_\alpha \sim$ half-StudentT(0, 26.7, 3) [prior for standard deviation of ID]

1.3. Prior predictive checking

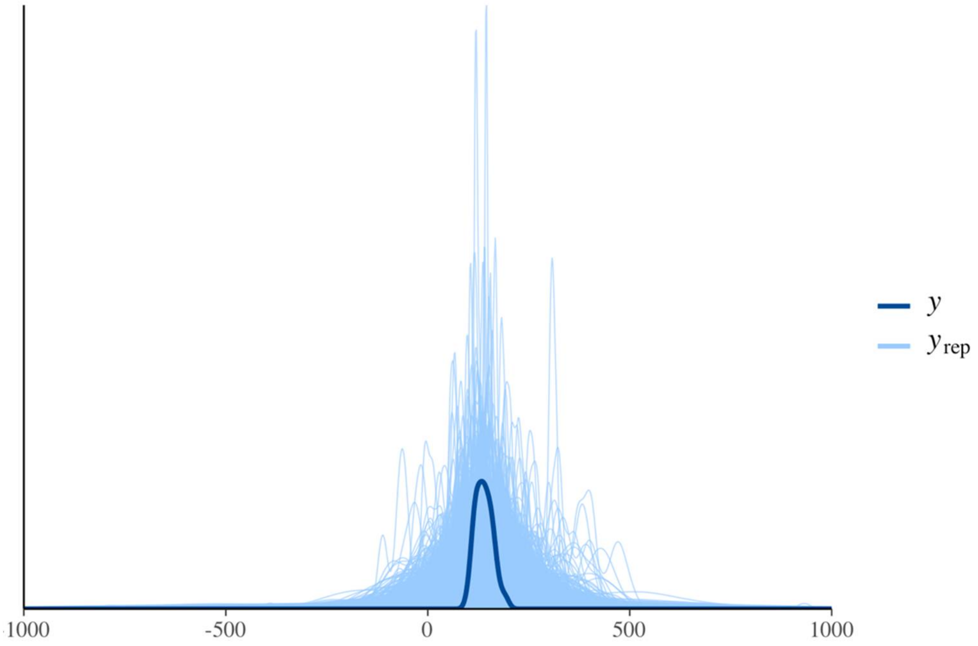

Figure S2. Graphical representation of the empirical distribution of the data (y) and simulated data from the prior predictive distribution (y_{rep}).

1.4. Parameter estimation and graphical diagnostics

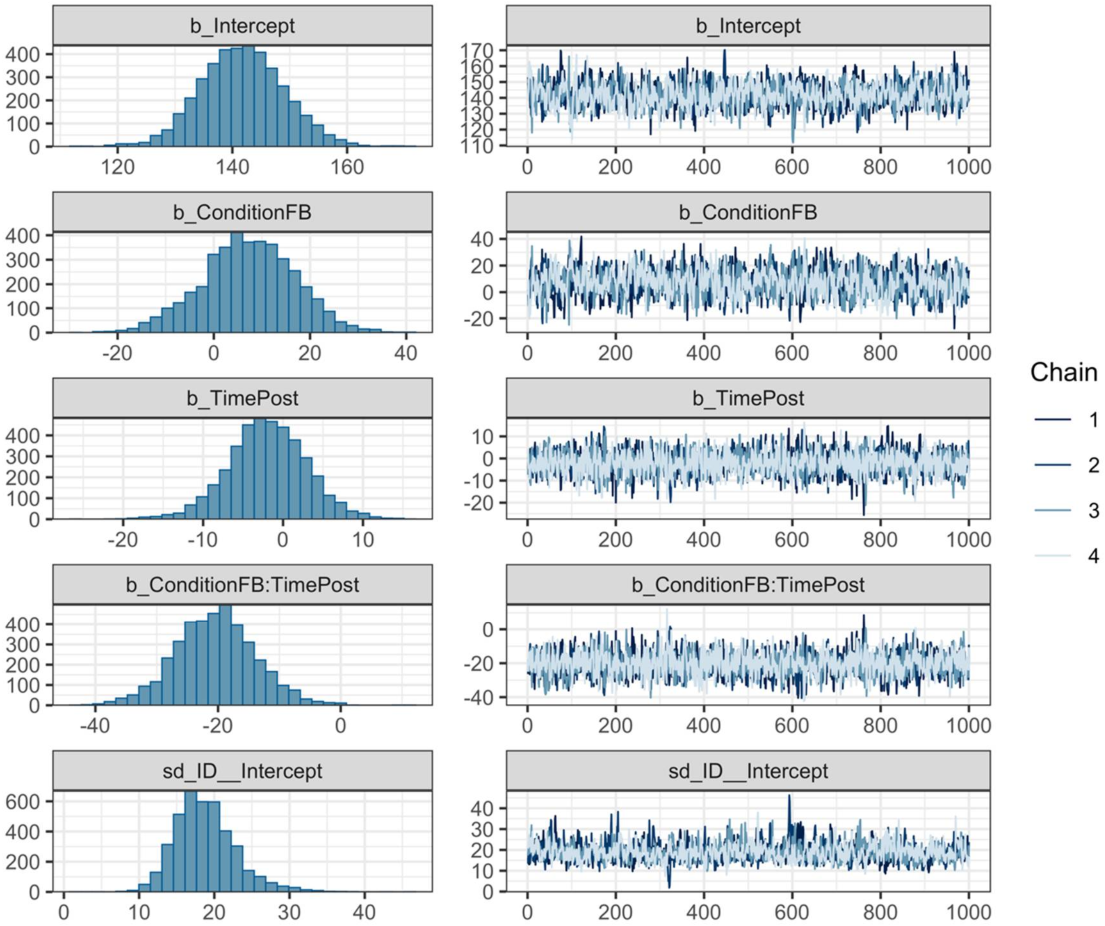

Figure S3. Left column represents histogram of each parameter estimated. Right column are traceplots of the four Markov chains.

1.5. Posterior predictive checking

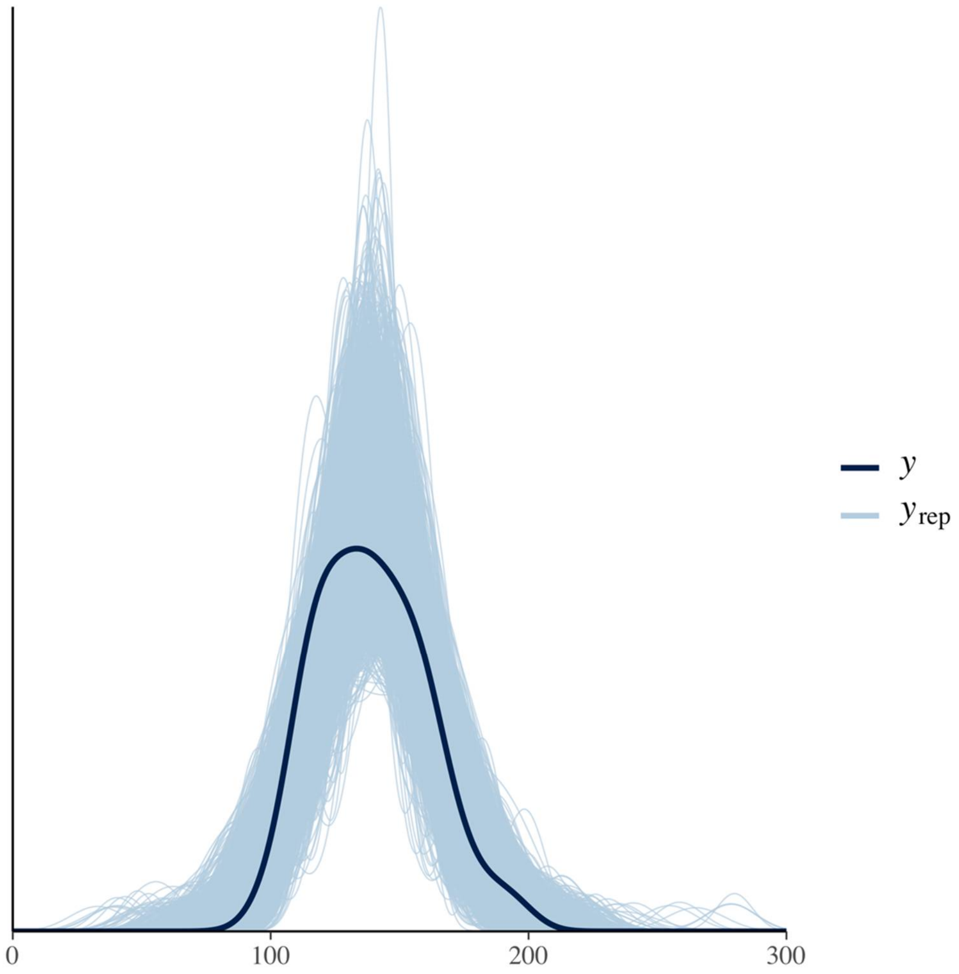

Figure S4. Graphical representation of the empirical distribution of the data (y) and simulated data from the posterior predictive distribution (y_{rep}).
